# Bilateral elastofibroma dorsi

**DOI:** 10.1590/0100-3984.2015.0137

**Published:** 2016

**Authors:** Juliana Pessoa, Aline Amaral Dal Sasso, Miriam Menna Barreto, Gláucia Maria Ribeiro Zanetti, Edson Marchiori

**Affiliations:** 1 Universidade Federal do Rio de Janeiro (UFRJ), Rio de Janeiro, RJ, Brazil.

*Dear Editor,*

**Figure 1:**
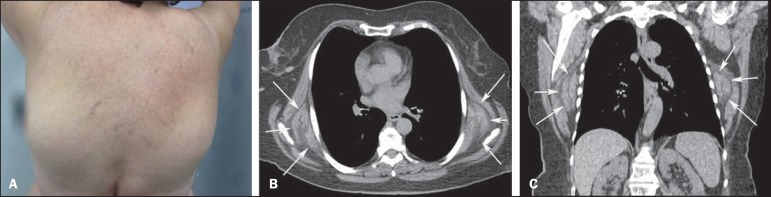
**A:** Photo of the patient’s dorsal region showing the appearance of infrascapular tumors. **B,C:** Computed tomography, axial (**A**) and coronal (**B**) sections showing bilateral, symmetrical masses in the infrascapular region (arrows).

A 76-year-old female patient with a previous history of surgically resected rectal cancer for seven years was admitted for diagnostic investigation of bilateral and symmetrical dorsal masses. She reported chronic pain in the thoracic spine. At clinical examination, the masses were solid, mobile, located subcutaneously and inferiorly to the scapulae (Figure 1A). Computed tomography (CT) showed the presence of bilateral soft tissue masses in the infrascapular region (Figures 1B and 1C). On the basis of the clinical findings and the images, the diagnosis of elastofibroma dorsi (ED) was established.

ED is a slow growing soft tissue pseudotumor incidentally diagnosed during routine imaging studies, that may also cause chronic scapular pain^(1)^. It is a benign fibroelastic tumor inferiorly located in the infrascapular region, between the scapula and the thoracic wall deeply to the serratus and latissimus dorsi muscles, possibly inserting into the periosteum of the posterior ribs. Coincidentally such type of tumor has been detected at CT in up to 2% of elderly patients^(1,2)^. It is most frequently found in elderly women (female to male ratio 5:1) in the age range between 65 and 70 years at the moment of the diagnosis^(3)^.

Unilateral masses are slightly more prevalent at the right side, but up to 60% of EDs are bilateral^(3)^. Other reported sites include deltoid muscle, axillae, ischial tuberosity, olecranon, hands and feet, among others^(4)^. It is also characterized by symptoms absence at early phases. With the disease progression, there is an increase in the mass volume, possibly limiting the upper limb motion, principally in the upward movements of the arm which require sliding of the scapula in relation to the thoracic wall. Such a movement may cause pain^(5)^. Macroscopically, ED is characterized by an ill defined mass with fibrous tissue and internal adipose tissue. Histopathological analysis demonstrates non-encapsulated hypocellular mass composed of benign fibroblasts, eosinophilic collagen bundles and apparently fragmented elastic fibers, with groups of interposed mature adipocytes^(1,3,6)^.

Although in most cases of thoracic investigation magnetic resonance imaging (MRI) is indicated to evaluate extrapulmonary lesions, and CT remains reserved for investigation of parenchymal diseases^(7-11)^, CT may be diagnostic in cases where the lesion presents as an infrascapular or subscapular ill defined, non-encapsulated soft parts mass, isoattenuating to the muscles (fibrous tissue), interspersed with fat attenuation strips or lines. Homogeneity may be observed in cases of smaller masses^(1,3)^. MRI is the method of choice for the diagnosis and demonstrates an expansile, solid, ill defined, non encapsulated and heterogeneous mass, with predominance of isosignal in relation to the muscles (fibrous tissue) and, typically, intermingled with hypersignal lines on T1- and T2-weighted sequences (fat tissue)^(1)^. Recently, reports about ED detection at positron emission tomography were published in the literature. Mild or moderate fluorodeoxyglucose uptake was frequently observed and should not be interpreted as a malignant finding^(2)^. In cases of asymptomatic lesions, there is no need for excision. Surgical resection in indicated in cases were pain and discomfort are present^(12)^.
